# Schizophrenia: a consequence of gene-environment interactions?

**DOI:** 10.3389/fnbeh.2014.00435

**Published:** 2014-12-23

**Authors:** Tim Karl, Jonathon C. Arnold

**Affiliations:** ^1^Neuroscience Research Australia (NeuRA)Randwick, NSW, Australia; ^2^Schizophrenia Research InstituteDarlinghurst, NSW, Australia; ^3^School of Medical Sciences, University of New South WalesSydney, NSW, Australia; ^4^Department of Pharmacology, Bosch Institute, University of SydneySydney, NSW, Australia; ^5^Brain and Mind Research InstituteCamperdown, NSW, Australia

**Keywords:** Schizophrenia, gene, environment, interaction, GxE, animal model, two-hit hypothesis

Schizophrenia is a multi-factorial disease characterized by a high heritability and environmental risk factors (e.g., social stress and cannabis use). It is the combined action of multiple genes of small effect size (Owen et al., 2005) and a number of environmental risk factors (McGrath et al., 2004), which causes the development of this mental disorder (Mackay-Sim et al., 2004). This is conceptualized in the “Two-Hit Hypothesis” of schizophrenia, which predicts that genetic and environmental risk factors interactively (GxE interaction) cause the development of the disorder (Bayer et al., 1999; Caspi and Moffitt, 2006). GxE interactions occur when the expression of an individual's genetic predisposition is dependent on the environment they are living in or when environmental influences on a trait differ according to an individual's genome (Tsuang et al., 2004). Human studies have confirmed that nature and nurture are both important in the development of schizophrenia: the concordance rate in monozygotic twins is only around 50% (Tsuang et al., 2001) and genome wide association studies fail to identify major genetic candidates for schizophrenia (Sanders et al., 2008) suggesting an important role of environmental factors in the development of this disorder.

Research into GxE is starting to produce valuable new animal models and has revealed novel insights into the pathophysiology of schizophrenia. This work will help advance our understanding of the molecular pathways involved in this mental disorder and will lead to more specific treatment avenues. Furthermore, understanding GxE interactions will guide the development of new preventative measures (i.e., people genetically predisposed to schizophrenia will be able to minimize exposure to critical environmental risk factors). However, these outcomes are a long way ahead and the complexity of this multi-factorial line of research has also caused difficulties in data interpretation and comparison. Thus, this research topic is intended to cover past and current directions in research dedicated to the understanding of GxE interactions in schizophrenia, the molecular, neurobiological, and behavioral consequences of these interactions, and potential mechanisms involved.

Animal models can incorporate highly standardized genetic and environmental risk factors at critical stages of brain development. These models thereby attempt to mimic the etiology of schizophrenia and help elucidate relevant interactions between GxE and the underlying mechanisms. In this Research Topic, Cash-Padgett and Jaaro-Peled outline how either early immune activation (i.e., PolyI:C treatment) or forms of social stress (i.e., social isolation, chronic social defeat) influence the development of schizophrenia-relevant phenotypes in a number of genetic mouse models for the schizophrenia susceptibility gene *Disrupted in schizophrenia-1* (*DISC1*). Their review concludes that animal models can be instrumental in linking the relevance of GxE interactions to a particular neuropsychiatric disorder. Furthermore, “environmental interaction profiles” can be developed for major risk genes such as *DISC1* (Cash-Padgett and Jaaro-Peled, 2013). Karl as well as Chohan and co-workers focus on the role the schizophrenia susceptibility gene *neuregulin 1* (*NRG1*) might play in the context of GxE interactions. Karl's review overviews a raft of evidence supporting *Nrg1* as a key player in the vulnerability of mice to environmental (risk) factors such as stress, cannabis abuse and housing conditions (Karl, 2013). Following on from recent published work (Chohan et al., 2014a), Chohan et al. describes research in which partial genetic deletion of *Nrg1* modulates the effects of adolescent stress on N-methyl-d-aspartate receptors (NMDAR), with the *Nrg1*-stress interaction tending to decrease NMDAR binding in the medial prefrontal cortex (Chohan et al., 2014b). This is significant as NMDAR is decreased in brains of schizophrenia patients and implicated in the pathophysiology of the disorder. In another research paper, Klug and van den Buuse investigate the effects of young-adult cannabinoid exposure on BDNF deficient mice and find no interaction in this GxE model on learning and memory later in life. Their research highlights that not all GxE models produce interactive effects and that the role of BDNF in conferring vulnerability to the actions of cannabis may be sex-dependent and only evident after pre-exposure to the drug (Klug and van den Buuse, 2013).

Jiang and coworkers provide an excellent review outlining the potential mechanisms behind established GxE interactions using mice with conditional knock down of NMDAR in cortical and hippocampal interneurons during early postnatal development. They suggest that NMDAR hypofunction and social isolation stress interact to disturb parvalbumin-positive (PV) interneurons via oxidative stress mediated by the transcriptional coactivator peroxisome proliferator activated receptor γ coactivator 1α (PGC-1α) (Jiang et al., 2013). Reductions in PV expression have been noted in schizophrenia brain and may help explain asynchrony of neuronal networks (Lewis and Gonzalez-Burgos, 2006). Showing that oxidative stress is responsible for PV interneuron dysfunction resonates with the recent interest in antioxidants such as fish oil as new treatments for mental disorder, particularly prior to disease onset. Extending on the idea of early intervention treatments as the key to treating schizophrenia is the research paper of Van Vugt and colleagues who show that a lack of early maternal care exacerbated schizophrenia-relevant phenotypes in a pharmacological model of the disorder (Van Vugt et al., 2014).

Girardi and coworkers also utilize maternal deprivation to investigate another aspect of the “Two-Hit Hypothesis” of schizophrenia, namely, environment x environment interactions (ExE). They report that maternally deprived rats are vulnerable to the effects of a very mild stress procedure (i.e., saline injection) on corticosterone levels and social behaviors (Girardi et al., 2014). This study is important for studies into GxE interactions, as researchers must be aware that even a relatively minor intervention such as an i.p. injection can modify the stress response in an established environmental model of schizophrenia. Thus, ExE (as well as GxExE) interactions exist and could have a significant impact on established GxE model systems. Within this context, Turner and Burne point out that housing conditions and genetic background both impact on cognitive behaviors of rodents. As cognition is an important domain for the evaluation of face validity of rodent models for schizophrenia, rodent strain and housing conditions must carefully be considered when developing novel model systems for GxE (Turner and Burne, 2013). The last two studies are in line with what is discussed by Burrows and Hannan. Their opinion paper outlines the need for more accurate and sophisticated GxE animal models of schizophrenia and the impact “unexpected” housing conditions can have on genetic mouse models. Environmental factors with clinical relevance should be manipulated to broaden the relevance of preclinical research beyond “standard laboratory housing” and to understand how a decanalized brain produces suboptimal phenotypes' (Burrows and Hannan, 2013).

Godar and Bortolato review evidence suggesting that gene x sex interactions (GxS) are also evident in schizophrenia as well particularly when focusing on genes involved in dopamine pathways. They outline that genes encoding enzymes that regulate dopamine levels, such as catechol-O-methyl transferase (COMT) and *monoamine oxidase*, are sexually dimorphic and the impact of sex hormones on their regulation may play a significant role in shaping the course of schizophrenia (Godar and Bortolato, 2014). This is particularly relevant seeing males tend to manifest the disorder earlier than females. Sexual dimorphism also influences GxE as highlighted above in the work of Klug and co-workers (Klug and van den Buuse, 2013) and as noted in various studies reviewed by Karl (2013) in this Research Topic. Importantly, COMT has been shown to be a good candidate for GxE interactions as the gene interacts with adolescent cannabis abuse (Caspi and Moffitt, 2006). Finally, Miller assessed the impact of photic cues on the development of schizophrenia. Her argument for a role for photic cues in the disorder is made more compelling given that single nucleotide polymorphisms have been found in genes relevant to schizophrenia, which are modulated by photoperiod and sunlight intensity (Miller, 2013).

GxE interactions appear to be complex and sensitive to a number of subtle variables, but do exist and justify the need for future research in this area (Van Os et al., 2010). Animal researchers should focus on models with significant relevance to schizophrenia such as cannabis abuse, maternal immunization, or early life stress and consider a number of genetic candidates for GxE interactions. Importantly, some of the articles of this topic suggest that valid GxE animal models will be very sensitive to the laboratory environment, which demands a high level of transparency and standardization of test conditions as well as thorough consideration of housing conditions across research sites. Furthermore, it should be mentioned that there are discussions about the appropriate statistical modeling of GxE interactions, which influence both animal and human research (Zammit et al., 2010).

To conclude, animal models have provided compelling evidence for GxE in schizophrenia based on the greater experimental control possible and the ability to manipulate specific genes and environmental factors. While such studies cannot reproduce the entire complexity of schizophrenia, they do provide simplified multifactorial models of aspects of the condition. These, in turn, allow new molecular and neurobiological insights to be gained and novel drug targets to be discovered.

## Conflict of interest statement

The authors declare that the research was conducted in the absence of any commercial or financial relationships that could be construed as a potential conflict of interest.

## References

[B1] Bayer T. A. Falkai P. Maier W. (1999). Genetic and non-genetic vulnerability factors in schizophrenia: the basis of the “two hit hypothesis”. J. Psychiatr. Res. 33, 543–548. 10.1016/S0022-3956(99)00039-4 10628531

[B2] Burrows E. L. Hannan A. J. (2013). Decanalization mediating gene-environment interactions in schizophrenia and other psychiatric disorders with neurodevelopmental etiology. Front. Behav. Neurosci. 7:157 10.3389/fnbeh.2013.00157 PMC3826253 24312026

[B3] Cash-Padgett T. Jaaro-Peled H. (2013). DISC1 mouse models as a tool to decipher gene-environment interactions in psychiatric disorders. Front. Behav. Neurosci. 7:113. 10.3389/fnbeh.2013.00113 24027503 PMC3759735

[B4] Caspi A. Moffitt T. E. (2006). Gene-environment interactions in psychiatry: joining forces with neuroscience. Nat. Rev. Neurosci. 7, 583–590. 10.1038/nrn1925 16791147

[B5] Chohan T. W. Boucher A. A. Spencer J. R. Kassem M. S. Hamdi A. A. Karl T.. (2014a). Partial genetic deletion of Neuregulin 1 modulates the effects of stress on sensorimotor gating, DENDRITIC MORPHOLogy, and HPA axis activity in adolescent mice. Schizophr. Bull. 40, 1272–1284. 10.1093/schbul/sbt193 24442851 PMC4193694

[B6] Chohan T. W. Nguyen A. Todd S. M. Bennett M. R. Callaghan P. Arnold J. C. (2014b). Partial genetic deletion of neuregulin 1 and adolescent stress interact to alter NMDA receptor binding in the medial prefrontal cortex. Front. Behav. Neurosci. 8:298. 10.3389/fnbeh.2014.00298 25324742 PMC4179617

[B7] Girardi C. E. Zanta N. C. Suchecki D. (2014). Neonatal stress-induced affective changes in adolescent Wistar rats: early signs of schizophrenia-like behavior. Front. Behav. Neurosci. 8:319. 10.3389/fnbeh.2014.00319 25309370 PMC4159973

[B8] Godar S. C. Bortolato M. (2014). Gene-sex interactions in schizophrenia: focus on dopamine neurotransmission. Front. Behav. Neurosci. 8:71. 10.3389/fnbeh.2014.00071 24639636 PMC3944784

[B9] Jiang Z. Cowell R. M. Nakazawa K. (2013). Convergence of genetic and environmental factors on parvalbumin-positive interneurons in schizophrenia. Front. Behav. Neurosci. 7:116. 10.3389/fnbeh.2013.00116 24027504 PMC3759852

[B10] Karl T. (2013). Neuregulin 1: a prime candidate for research into gene-environment interactions in schizophrenia? Insights from genetic rodent models. Front. Behav. Neurosci. 7:106. 10.3389/fnbeh.2013.00106 23966917 PMC3744031

[B11] Klug M. van den Buuse M. (2013). An investigation into “two hit” effects of BDNF deficiency and young-adult cannabinoid receptor stimulation on prepulse inhibition regulation and memory in mice. Front. Behav. Neurosci. 7:149. 10.3389/fnbeh.2013.00149 24155701 PMC3800788

[B12] Lewis D. A. Gonzalez-Burgos G. (2006). Pathophysiologically based treatment interventions in schizophrenia. Nat. Med. 12, 1016–1022. 10.1038/nm1478 16960576

[B13] Mackay-Sim A. Feron F. Eyles D. Burne T. Mcgrath J. (2004). Schizophrenia, vitamin D, and brain development. Int. Rev. Neurobiol. 59, 351–380. 10.1016/S0074-7742(04)59014-1 15006495

[B14] McGrath J. Saha S. Welham J. El Saadi O. Maccauley C. Chant D. (2004). A systematic review of the incidence of schizophrenia: the distribution of rates and the influence of sex, urbanicity, migrant status and methodology. BMC Med. 2:13. 10.1186/1741-7015-2-13 15115547 PMC421751

[B15] Miller C. L. (2013). Evidence for phenotypic plasticity in response to photic cues and the connection with genes of risk in schizophrenia. Front. Behav. Neurosci. 7:82. 10.3389/fnbeh.2013.00082 23847488 PMC3705146

[B16] Owen M. J. Craddock N. O'donovan M. C. (2005). Schizophrenia: genes at last? Trends Genet. 21, 518–525. 10.1016/j.tig.2005.06.011 16009449

[B17] Sanders A. R. Duan J. Levinson D. F. Shi J. He D. Hou C.. (2008). No significant association of 14 candidate genes with schizophrenia in a large European ancestry sample: implications for psychiatric genetics. Am. J. Psychiatry 165, 497–506. 10.1176/appi.ajp.2007.07101573 18198266

[B18] Tsuang M. T. Bar J. L. Stone W. S. Faraone S. V. (2004). Gene-environment interactions in mental disorders. World Psychiatry 3, 73–83. 16633461 PMC1414673

[B19] Tsuang M. T. Stone W. S. Faraone S. V. (2001). Genes, environment and schizophrenia. Br. J. Psychiatry Suppl. 40, s18–s24. 10.1192/bjp.178.40.s18 11315219

[B20] Turner K. M. Burne T. H. (2013). Interaction of genotype and environment: effect of strain and housing conditions on cognitive behavior in rodent models of schizophrenia. Front. Behav. Neurosci. 7:97. 10.3389/fnbeh.2013.00097 23914162 PMC3728474

[B21] Van Os J. Kenis G. Rutten B. P. (2010). The environment and schizophrenia. Nature 468, 203–212. 10.1038/nature09563 21068828

[B22] Van Vugt R. W. Meyer F. Van Hulten J. A. Vernooij J. Cools A. R. Verheij M. M.. (2014). Maternal care affects the phenotype of a rat model for schizophrenia. Front. Behav. Neurosci. 8:268. 10.3389/fnbeh.2014.00268 25157221 PMC4128220

[B23] Zammit S. Owen M. J. Lewis G. (2010). Misconceptions about gene-environment interactions in psychiatry. Evid. Based Ment. Health 13, 65–68. 10.1136/ebmh1056 20682811

